# An exploratory review of investments by development actors in health workforce programmes and job creation

**DOI:** 10.1186/s12960-023-00835-3

**Published:** 2023-07-07

**Authors:** Remco van de Pas, Linda Mans, Myria Koutsoumpa

**Affiliations:** 1 Institute of Tropical Medicine, Department of Public Health, Nationalestraat 155, 2000 Antwerp, Belgium; 2 Centre for Planetary Health Policy, Cuvrystrasse 1, 10997 Berlin, Germany; 3 Manskracht, Van den Havestraat 42, 6521 JS Nijmegen, The Netherlands; 4 Wemos, Plantage Middenlaan 14, 1018 DD Amsterdam, The Netherlands

**Keywords:** Health workforce, Global strategy, World Health Organization, Investment, Development cooperation, Governance, Fiscal space, Covid-19, Health employment, Economic growth

## Abstract

The World Health Organization’s Global Strategy on Human Resources for Health: Workforce 2030 identified a projected shortfall of 18 million health workers by 2030, primarily in low- and middle-income countries. The need for investment was re-enforced by the 2016 report and recommendations of the United Nations High-Level Commission on Health Employment and Economic Growth. This exploratory policy tracing study has as objective to map and analyse investments by bilateral, multilateral and other development actors in human resources for health actions, programmes and health jobs more broadly since 2016. This analysis will contribute to the accountability of global human resources for health actions and its commitment by the international community. It provides insights in gaps, priorities and future policies’ needs. The study follows an exploratory rapid review methodology, mapping and analysing the actions of four categories of development actors in implementing the ten recommendations of the United Nations High-Level Commission on Health Employment and Economic Growth. These four categories of actors include (A) bilateral agencies, (B) multilateral initiatives, (C) international financial institutions and (D) non-state actors. Analysing the data generated via this review, three trends can be observed. Firstly, while a broad range of human resources for health actions and outputs have been identified, data on programme outcomes and especially on their impacts are limited. Secondly, many of the programmatic human resources for health actions, often funded via bilateral or philanthropic grants and implemented by non-governmental organisations, seemed to be rather short-term in nature, focusing on in-service training, health security, technical and service delivery needs. Despite the strategic guidance and norms developed by multilateral initiatives, such as the International Labour Organization–Organisation for Economic Co-operation and Development–World Health Organization Working for Health programme, has it been for several development projects difficult to assess how their activities actually contributed to national human resources for health strategic development and health system reforms. Lastly, governance, monitoring and accountability between development actors and across the policy recommendations from the United Nations High-Level Commission on Health Employment and Economic Growth could be improved. There has been limited actionable progress made for the enablers required to transform the workforce, including in the domain of generating fiscal space for health that would strengthen jobs in the health sector, the development of health workforce partnerships and its global agenda, and the governance of international health workforce migration. In conclusion, one can observe that global health workforce needs are much recognised, especially given the impact of the Covid-19 pandemic. However, 20 years after the Joint Learning Initiative on Human Resources for Health, there is still an urgent need to take shared responsibility for international cooperative action for overcoming and addressing persistent underinvestment in the health workforce. Specific policy recommendations are provided to this end.

Policy recommendations for international health workforce investments by development actors

1. Develop a truly coherent global health workforce financing agenda and collaborative framework, including and beyond development aid for low- and middle-income countries. Solidarity and cooperation at the multilateral, regional and domestic level are required to sustain health workforce investments.
2. Address the health skills supply gap of the global market by providing financial and non-financial incentives to middle- and high-school graduates to enter health professions.
3. Significantly increase Official Development Assistance funding via investments in creating health sector jobs for youth and women.
4. Create sustainable health labour mobility partnerships between countries.
5. Extensive debt cancellation and the issuing of more Special Drawing Rights by the International Monetary Fund; reforms of international corporate taxation; creation of a new multilateral health systems financing mechanism; and reforming the conditions tied to external loans and their impact on fiscal space are necessary macro-economic policy enablers that supplement domestic finance and development aid in rapidly scaling up health workforce investments.

## Background

The World Health Organization (WHO) has argued in its Global Strategy on Human Resources for Health: Workforce 2030 for much needed investments in health and health systems as to meet the future needs of populations [1]. A systematic analysis in 2017 estimated that an additional US$ 92 billion to US$ 150 billion would be needed annually to help strengthen the health and care workforce (HCWF) in Low- and Middle-Income Countries (LMICs) [2]. Many countries will thus continue to need external financial support throughout the period of the Sustainable Development Goals (SDGs), mostly to develop the foundations of their health systems. However, only 7% of all development assistance for health went to support health workforce between 1990 and 2020 [3].

This exploratory policy tracing study has the objective to map and analyse investments by bilateral, multilateral and other development actors in human resources for health (HRH) actions, programmes and health jobs more broadly since 2015. This analysis will contribute to the accountability of global HRH actions and its commitment by the international community. It provides insights in gaps, priorities and future policies’ needs. It will aim to answer the following research questions:

1. What have been the major actors, domains and impact of HRH actions and policy that have been funded via bilateral, multilateral and other development funding since 2015?
2. To what extent have these investments been leveraged, or hampered by, financing policies at national and international level?
3. What kind of development recommendations can be elicited for future HRH investment and needs, also given the Covid-19 policy momentum?

## Methodology

Given the considerable variety and contextual factors of different bilateral, multilateral and other HRH interventions, the study follows an exploratory rapid review methodology, applying an iterative and snowballing search strategy of academic literature via Google Scholar, Web of Science, policy documents, websites of governments, non-governmental organisations (NGOs), United Nations (UN) agencies and other actors involved, as well as other secondary data that provide insight in qualitative aspects of development cooperation for HRH.

The generic search terms included the main terms of the four strategic objectives of workforce 2030, as well as the ten recommendations by the United Nations High-Level Commission on Health Employment and Economic Growth (UNHEEG). These were matched with the terms ‘Official Development Assistance (ODA)’, ‘development cooperation’, ‘development partners’, ‘bilateral cooperation’, ‘multilateral agencies’, ‘international investment’, ‘international financial institutions’, ‘NGO’ as well as the specific names of several actors known to be active in health workforce development cooperation.

The ensuing categorisation followed characteristics of the development actors and financial flows involved. Four categories were applied, representing a broad set of actors considered most relevant for international cooperation on health workforce development:

A. Bilateral state-to-state funded cooperation, including the non-state actors and programmes funded through this collaboration;
B. Multilateral cooperation, which includes cooperation with UN institutions, Organisation for Economic Co-operation and Development (OECD), regional entities like European or African Union and likewise via the ‘hybrid’ Global Health Initiatives such as the Global Fund to Fight AIDS, Tuberculosis and Malaria (GF), Gavi the Vaccine Alliance (Gavi), etc.;
C. Cooperation via International Financial Institutions (IFIs) and Development Banks, such as the International Monetary Fund (IMF) and World Bank (WB);
D. Other non-state actor collaborations, which include philanthropy funded and private sector cooperation, as well as partnerships between health professional, education or NGO groups, and broader civil society actions.

These categories, and actors involved, are not mutually exclusive. For the purpose of this study, we have iteratively selected the main actors relevant for HRH development cooperation, hereby excluding several smaller cooperative partnerships that were less financed and documented. The four categories provide the structure to analyse and compare the different HRH actions and funding mechanisms.

These four actor categories and their programmes were consecutively organised in a matrix against the ten objectives, “To transform the health workforce for the SDGs” as spelled out by the UNHEEG [4]. These ten objectives incorporate to a large degree the four objectives of Workforce 2030 into a broader inter-sectoral framework for SDGs implementation from a health workforce perspective. Out of these ten objectives, six focus on the change required and four on the enablers of this change (see Table 1). These UNHEEG objectives are listed in the results section in Italic as (*UNHEEG Nr).*

**Table 1:** Overview of the ten UNHEEG objectives

*Six objectives focus on change needed in health employment, health education and health service delivery*
1. Job creation
2. Gender and women’s rights
3. Education, training and skills
4. Health service delivery and organisation
5. Technology
6. Crisis and humanitarian settings
*Four objectives focus on how to enable the necessary changes*
7. Financing and fiscal space
8. Partnership and cooperation
9. International migration
10. Data, information and accountability

Data inclusion focused on actions taken after 2015. We specifically traced programmes, finance and type of actions undertaken by different actors. We also checked whether possible outcomes and impacts were reported. Actions taken by national governments without a direct international cooperation aspect were excluded from this analysis. Likewise, collaborations with a specific intra-regional focus were excluded for the main reason that the financial volume involved was often limited.

While the type of bilateral/multilateral finance and actions have not been applied rigidly, it would in general adhere to ODA criteria. The review looked for specific actions on health workforce cooperation. Broader collaborative actions supporting health systems strengthening (HSS), advancing Universal Health Coverage (UHC), improving generic public sector jobs, reform and education, humanitarian relief, health emergency programmes and broader labour migration programmes have been excluded from this search. Likewise, a range of health workforce programmatic collaborations that are relevant but small in expenditure, e.g., below US$ one million per annum, have been excluded from the dataset given their limited overall impact. English, French and Spanish sources have been included. Other languages have been excluded given language competency of researchers.

The data collected have been extracted and organised in an excel sheet, categorising type of actors across the ten UNHEEG recommendations. Further categorisation included type of funding sources, a brief overview of actions, countries and regions of implementation, timeline and outcome. The concept of fiscal space, and the analysis on why collaboration on this matters for health workforce collaboration, is presented.

A separate study quantifying HRH actions and expenditure via ODA is published in this special issue. This qualitative review builds on and complements the analysis and recommendations presented there [3].

The researchers have made use of the existing datasets and analysis conducted in tracing the policy actions of governments and development partners after commitments made during the third global forum on HRH in 2013 [5] as well as the data collected for the independent review of the Working for Health (W4H) programme in 2021 [6].

In total, 19 academic articles as well as 55 programme reports, policy analyses and websites have been reviewed and included in the dataset.

## Results

**Fig. 1:**
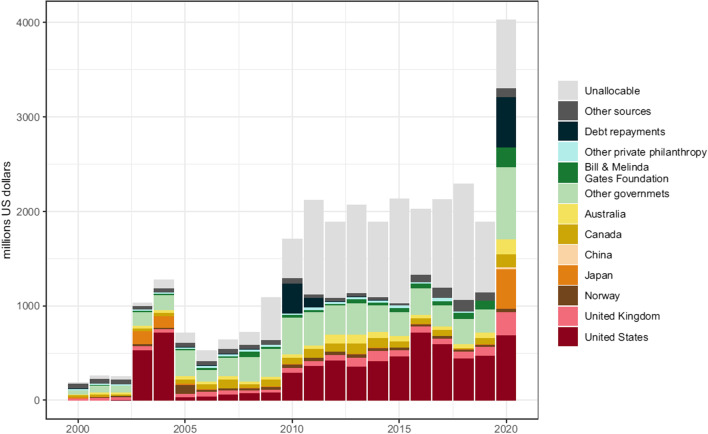
Official development assistance for human resources for health by source of funding; millions of 2020 US$ [3].

Our qualitative analysis matches the available quantitative analysis on HRH expenditure from ODA [3]. Given the relevance of this study, the reproduced figure and explanation below provide a quantitative overview of HRH expenditure by source of ODA funding (Fig. 1).

This figure indicates an absolute increase in ODA funding for HRH since early 2000s, while as a proportion of overall ODA for health it has remained stable at about 5–7% [3]. Over the last decade ODA for HRH has been provided by a small group of donor countries, notably the United States of America (USA), the United Kingdom (UK), Japan and Canada. The share of ‘other governments’ is likely coming from several European countries. Debt repayments and the boost to HRH funding is likely related to the Muskoka Initiative and fund on Maternal, Newborn and Child Health in 2010 [7]. The growing share of unallocable funding is probably due to expenditure by global health initiatives and multilaterals trust funds such as the GF, Gavi, Global Financing Facility (GFF), the W4H program and NGOs [3]. The proportion of funding by philanthropic organisations including the Bill & Melinda Gates Foundation has slightly increased over the recent years. The Covid-19 pandemic led to a considerable increase in short-term HRH spending, including via debt repayments, as to contain and respond to this global health crisis. It is to be seen if and how this increase will be sustained.

A qualitative description of HRH policy action per actor group is provided in the results section below. Given its importance in leadership, norm-setting and coordination, we first outline the overarching role of the WHO.

### The World Health Organization

WHO has been the main actor and coordinator of the W4H programme (which also includes the International Labour Organization (ILO) and OECD) following the UNHEEG report from 2016. Besides being the lead agency responsible for governance and accountability, its specific contributions are outlined below.

WHO has leveraged on the decent employment theme by supporting multisectoral technical capacity in countries on the application of national health labour market analysis to develop policy options and actions. With W4H funding, this has been initiated in 16 countries, mainly in sub-Sahara Africa (SSA), Asia and the Middle East. This is supported by a health labour market analysis guidebook, developed by WHO [8]. In addition, ILO and WHO jointly provide guidance and assistance to countries to develop and implement occupational health and safety programmes for health workers, of which ‘Caring for those who care’ is an example [9]. The W4H programme has an important role in aligning international cooperation and investments with national health, labour and education strategies. W4H has focused on a number of SSA countries, like Guinea, Niger and South Africa as well as worked with regional authorities on economic development and health employment in the Western and Southern African region. The W4H programme enabled coordination between several ministries, international development partners, and professional associations on HRH policy action [6].

WHO is ramping up new E-Health learning modalities, via the establishment of the new WHO Academy, which has the aim to stimulate lifelong learning for health professionals [10]. Moreover, new skills development as well as protection measures are required to enable health workers to continue their services in times of (health) crisis and humanitarian contexts *(UNHEEG 6).* WHO’s surveillance system for Attacks on Health Care is active since 2017 and provides an updated overview of violence directed against health care facilities and personnel, including on the type of violence and location of the event [11].

The domain of International Migration Cooperation (*UNHEEG 9*) has been an important area for cooperation of the WHO with non-state actors given the involvement of regulators, trade unions, health service employers, recruitment agencies and others. In 2018, WHO, together with OECD and ILO, set up the International Platform on Health Workforce Mobility (IPHWM) to advance dialogue, knowledge and cooperation in the area, notably to implement the WHO Global Code of Practice on the International Recruitment of Health Personnel [12]. This multi-actor platform facilitated an Expert Advisory Group to review the Relevance and Effectiveness of the WHO Global Code [13]. Complementarily, the IPHWM has aimed to implement the principles of the UN Global Compact for Safe, Orderly and Regular Migration [14]. Unfortunately, the IPHWM has not assembled since the start of the pandemic. WHO has published the health workforce support and safeguard list of 47 countries with the most pressing UHC-related health workforce challenges, and discourages active recruitment from these member states [15]. In 2023, the list was updated and the number of countries has increased to 55 [16].

### Bilateral cooperation for health workforce development

#### Education and training

In the domain of health workforce education, skills and training* (UNHEEG 3)* persistent bilateral cooperation has been evident. Organisations like IntraHealth International and THET have worked with American and British development cooperation funding, since more than a decade, in several SSA and Asian countries. They have provided collaborative programmes with both government and private actors to strengthen matters like workforce management, performance and capacity development. Between them, these organisations claimed to have trained over half a million of formal and community health workers over the last decade [17, 18]. Given a greater focus on global health security since the Ebola outbreak in West Africa in 2014–2016 [19], several bilateral agencies have supported training for health workers in surveillance, infectious prevention control, vaccination programmes and occupational safety. Complementarily, a wide range of programme activities and development cooperation projects have focused on improving health service delivery. E.g., Amref Health Africa, which receives support via bilateral donors and non-state actors, is working in a number of African countries on leadership, management and productivity of (community) health workers (*UNHEEG 4).* The organisation has advocated for the need to re-engineer and invest in the health workforce in SSA [20]. Bilateral donors like the United States Agency for International Development (USAID), the Japan International Cooperation Agency (JICA), and the Swedish International Development Cooperation Agency (SIDA) have implemented several health services and HRH development programmes, with different foci, but often including maternal and child health components, and cooperating with government and other partners in particularly the SSA and Central Asian regions. Similarly, the French Muskoka Fund aims to reduce maternal, newborn, infant and child mortality by strengthening national health systems, notably by investing in the health workforce [21]. Denmark joined the initiative at the end of 2018. The Muskoka Fund supports the joint work of four UN agencies (WHO, UN Women, United Nations Population Fund and UNICEF) and operates in nine countries in West and Central Africa [21].

#### Digital health

Since 2015, there has been a steady expansion of different digital health-services, education and information platforms (*UNHEEG 10*), supported by development actors, that enable knowledge sharing and interactions between health workers, patients and other relevant actors. USAID has financed, via several implementing agencies, the roll-out out of iHRIS Health Workforce Information Systems Software, an open-source software that tracks and manages health workforce data to improve access to services [22]. The HRH2030 programme implemented by Chemonics International has focused during several years on the data-integration and management of iHRIS across multiple sectors with the aim to improve governance of national health labour markets [23]. This data-integration approach is complemented by the technological application *(UNHEEG 5)* of “mHero”, which is a two-way, mobile, phone-based communication system that connects ministries of health and health workers. It has so far been implemented in seven countries [24]. Amref Health Africa has also over the years introduced and scaled-up digital innovations via E-Health applications and M-Health interventions in the maternal, newborn and child health domain of (community) health workers [25].

#### Covid-19

The Covid-19 pandemic triggered many bilateral actions and support, notably because of WHO’s International Year of Health and Care Workers campaign [26]. Despite the many workforce interventions that were initiated during the Covid-19 pandemic, it is difficult to have a qualitative, comprehensive, tracing of actions that took place. Most of these seem to have focused on short-term training activities for, and contracting of, health professionals and community health workers in providing emergency care and vaccinations during the pandemic but with little impact on financing sustainable health systems interventions. Regardless, relevant insights have emerged from the Covid-19 response. Many of the OECD countries already reliant on foreign nurses and doctors, have further recognised them as key assets, and implemented additional policy measures to ease their entry and the recognition of their professional qualifications [27]. Also, Covid-19 has helped making visible the vital professions of, amongst others, healthcare workers and that states can prioritise, even if briefly, health over wealth [28]. Decent work will be important in the Covid-19 economic recovery [29] and applying a gender-equity lens remains highly pertinent [30].

### Multilateral cooperation and health workforce development

#### Decent employment and job creation

The need for investment in decent employment and job creation *(UNHEEG 1)* had been re-enforced by the 2016 report and recommendations of the UNHEEG [4]. It formed the basis for the five-year action plan for health employment and inclusive economic growth and the related ILO–OECD–WHO W4H programme [31]. The W4H programme is financed via a Multi-Partner Trust Fund, which is managed by the United Nations Development Programme (UNDP). A review noted the programme being highly relevant, especially in light of the impact of the Covid-19 pandemic. However, the overall effectiveness of the W4H programme has been limited with about US$ 10 million in funding received by the Norwegian Agency for Development Cooperation (Norad), the Silatech foundation, and the UN Peace and Development Fund, while its intended five-year action plan target has been US$ 70 million [6].

The 2015 Addis Ababa Action Agenda on financing Sustainable Development stipulates that, besides low-income countries (LICs), the financing of public sector jobs is a national responsibility, to be generated via domestic resource mobilisation [32]. This principle, matched with the focus of macro-economic fiscal sustainability, implies that there is limited scope for development and multilateral partners to directly employ health workers. However, remuneration top-ups for public sector health workers are frequently applied in programmes. A 2017 review found that in 27 LICs, multilateral agencies and development banks played an important, technical and financial, role in advancing HRH actions [5].

#### The global health initiatives

The GF still employs, in a limited manner and only in LICs, health workers for its programmes. Though this seems considerably lower in all-over finance commitments than during the height of the HIV pandemic 15 years ago [33]. For instance, the GF has expended US$ 1.6 billion over the period 2003–2017 in 13 Eastern Mediterranean countries. The average expenditures allocated to “direct” HRH activities such as salaries, training costs, and technical assistance had been only 13% of total budgets [34].

During the pandemic, the GF focused on the provision of medical Personal Protective Equipment (PPE) for health care workers [35]. Investing in health workforce recurrent costs is discouraged for Gavi and the GFF (the latter being a WB Trust fund). Gavi is encouraging innovative HRH trainings in its grants. The GFF does not have yet a separate strategy on financing HRH in its grants but aims to be flexible in the wake of the pandemic with its HRH budgetary support. In fact, at its 14th Investors Group meeting in June 2022, the GFF recognised the need for strengthening HRH and decided to develop an operation plan to identify its role in supporting countries, which was endorsed in November 2022 [36]. It is suggested that Gavi, GF, and the GFF started pooling funds nationally around health system and workforce strengthening, allowing flexibility of their use, improving coordination among them and other agencies, and aligning with sound national HRH planning [37]. Nevertheless, HRH remains one of the neglected areas in The Global Action Plan for Healthy Lives and Well-being for all which brings together 13 multilateral health, development and humanitarian agencies to better support countries to accelerate progress towards the health-related SDGs [38].

#### Gender and data exchange

There are several examples of multilateral programmes that advocate for gender equality and women’s rights *(UNHEEG 2)* in relation to labour market inclusivity as well as the need to develop a gendered lens to health workforce investment and development. Studies, such as the recent joint ILO and WHO gender pay gap report, which indicates that women in the health and care sector earn 24% less than men, have made the call for large-scale investment to redress the undervaluation of the sector [9]. Generation Equality Forum (UN Women) [39] and the Muskoka G8 initiative [7] also note the need to invest with a gendered lens. In addition to this, many professional agencies, funders, UN bodies and civil society speak out, and address, on gender equality, jobs investment and reforms needed in the nursing and midwifery workforce [40].

The OECD, as part of the W4H programme, has developed together with WHO and ILO an Interagency Data Exchange which has contributed to the strengthening of the National Health Workforce Accounts (NHWA) database, which informs accountability for country actions. Health workforce stock data are available for 175 Member States for the top five occupations. Data on active foreign-trained workers are also available for 120 countries in the NHWA platform [41]. The OECD has further developed its health workforce statistics as part of its health systems monitoring work [42]. Furthermore, at WHO regional levels HRH development and policy is monitored or analysed via health workforce and systems observatories that provide detailed understanding of regional workforce development [43–45].

### Contributions by international financial institutions

#### Financing and fiscal space

IFIs have an important role in enabling the change recommendations of the UNHEEG, as international financing can be used to support catalytic investments in developing human capitals and skills for the health economy [46]. Actions on enabling financing and fiscal space *(UNHEEG 7*) for health employment, at an international and multilateral level, have been below the expectations expressed by the UNHEEG commission. With health workforce development and employment being considered (mainly) a domestic responsibility international investments in and technical support for increasing domestic revenues to this aim have been limited. Several LMICs have invested in their health workforce since the adoption of the global Workforce 2030 strategy [5]. Nevertheless, one sees a general global trend of adjustment and austerity in public expenditures over the decade 2010–2019, which is likely to be worsened in the years following the Covid-19 pandemic [47].

The WB has recommended five ways to expand fiscal space for health specifically in LMICs: economic growth, budget prioritisation, earmarking of certain revenues, improved efficiency of spending in health and external resources [48]. For instance, an analysis of 20 countries in East and Southern Africa indicated that the cumulative potential budget space for HRH could increase by 7.6% per annum up to 2026 if HRH is sufficiently prioritised within the health expenditure [49].

The IMF can also affect fiscal space for health, national policy-making, and the ability of a country to allocate resources into training, hiring, and retaining HRH via its fiscal and monetary policies. The IMF has been criticised for its emphasis on macro-economic stability and government solvency over social needs and development from increased public spending [50]. Research on conditions attached to IMF loans in 26 country programmes approved in the years 2016 or 2017 revealed that, contrary to what the IMF has been formally propagating, the majority were geared towards fiscal consolidation and wage bill freezes for public sector jobs, including in the health sector [51, 52]. Of the 57 countries identified by the WHO as facing critical health worker shortages, 24 received advice from the IMF to cut or freeze public sector wages in the three years before the pandemic [53].

#### Human capital

Although the WB made a shift in 2018 with its ‘Human Capital Project’, which has the aim to support countries to invest in people, and improve their human capital potential, the overall expectation that there is a reformulation of WB and IMF policies in the wake of the pandemic seems uncertain. The WB mentioned in 2021 that they are open for ‘HRH business’ in responding to the urgent workforce needs expressed during the Covid-19 pandemic [54]. The WB has provided US$ 12 billion in loans and grants to LMICs for purchasing vaccines. 20–30% of these funds should go to Community Health Workers supporting the roll-out of these vaccines [54]. These funds could in principle be used for the remuneration of health care workers. However, a NGO research report stated that out the 71 WB Covid-19 country projects, two-thirds of them do not include any plans to increase the number of health workers, and that the 25 projects which do, have substantial shortcomings, such as there being only temporal support for extra health workers, and no specification of the number of additional workers to be supported [55].

In 2020, WHO and the European Investment Bank (EIB) enhanced cooperation to support countries in addressing the health impact of Covid-19. This was followed in 2022 by a pledge of the EIB to make available at least €500 million to support HSS and more specifically Primary Health Care (PHC) in SSA- countries [56]. While there have been some vocational training programmes for health professionals funded by regional development banks in Asia and Africa, especially in relation to Covid-19 pandemic response, these did not constitute a main strategic theme for economic investment in the region.

The WB is amongst the financers of programmes to improve digital skills of health workers, whose use has expanded during the pandemic, [57] and that builds on WHO’s discussion paper on digital education for building health workforce capacity [58].

### Collaborations with non-state actors

#### Cooperation with NGOs, professional associations and the private sector

Non-state actors such as civil society, professional associations, philanthropic foundations and private commercial actors take an important role as collaborators in taking the objectives from the Workforce 2030 strategy and the UNHEEG action plan forward. The partnership and cooperation part *(UNHEEG 8)* refers to ‘aligning international cooperation to support investments in the health workforce, as part of national health and education strategies’ [4]. This approach got traction given the need to develop resilient health systems in the wake of the Ebola epidemic in West Africa (2014–2016) [59]. THET’s partnership model deserves mentioning as in the last decade it has reached over 100 000 health workers with training and education across 31 countries in Africa and Asia in partnership with over 130 UK institutions, including National Health Service (NHS) Trusts, Royal Colleges and academic institutions. This is funded through bilateral government cooperation grants, but includes a considerable degree of volunteer commitment [18]. The GF announced in 2022 a partnership with the Africa Frontline First Initiative to create a Catalytic Fund that will accelerate scale up of 200 000 community health workers deployed in ten SSA countries by 2030. The GF matches hereby private sector investments to the Catalytic Fund from philanthropic foundations totalling US$ 25 million [60]. This is an example of ‘blended finance’.

WHO has worked in close partnership with the International Council of Nurses, as well as the International Confederation of Midwives, to produce the State of the World Nursing report [61] and State of World’s Midwifery report [62], respectively. Both reports stress the chronic limitations of fiscal space in low-income and conflict affected countries and for the need to invest in education and employment for nursing and midwifery staff. Both reports argue that in these settings there is need for human capital investments and institutional fund-pooling arrangements by development partners and IFIs [61, 62].

#### Global Skills Partnerships

The Global Skills Partnerships approach as promoted by the Centre for Global development [63] has been applied in the health sector by bilateral organisations like the German Development Cooperation agency (GIZ). It has facilitated skills development and mobility partnerships for health care employment between German health care actors and labour migrants from the Philippines, Tunisia, and Kerala, which led to the recruitment of nearly 5000 professionals [64]. The WB is also much interested in this approach, i.e. for the recruitment of Nigerian nurses working in the UK health care system [65]. However, researchers have raised questions about the sustainability of these programmes [66]. An alternative health labour migration approach is the AHEAD project managed by Wemos that addresses, via a dialogue on policy reforms, health worker shortages in isolated or depopulated areas of the European Union and its neighbouring countries, known as ‘medical deserts’ [67].

Besides the cooperation in the workforce mobility domain, there is a considerable number of, often smaller in size but sometimes larger, partnerships, whereby states, private actors and philanthropy are involved and funded via ODA or other schemes. An observation here is that these initiatives are rather scattered, and that there is no real coordination, coherence or learning between them. This may be related to the Global Health Workforce Alliance (GHWA) ending its ten-year mandate in 2015, as there was too little donor interest in continuing funding this global partnership hosted at WHO [68]. Despite the existence of a loosely organised but limitedly funded Global Health Workforce Network (GHWN) that continued after the closure of GHWA [69] there is not, at the moment, an overarching collaborative form of networked partnership that could influence and maintain the health workforce action agenda at the global level.

## Discussion

The results provide an overview of HRH actions by developments actors, categorised across programmatic work by WHO, four actor categories, and the ten recommendations of the UNHEEG. It indicates that HRH programmatic action and its relevance for HSS, health security and economic development have been furthered since the adoption of the Workforce 2030 strategy. The majority of actions took place in the 55 countries covered by WHO in its health workforce support and safeguard list, with a considerable amount of these actions in SSA countries [16]. Three observations can be made analysing the data and comparing the four actor categories.

### A need for sustainable and integrated workforce cooperation

Firstly, many of the programmatic HRH actions, often funded via bilateral or philanthropic grants and implemented by NGOs, seem rather short-term in nature, focused on education, vaccination skills, technical and service delivery needs. Part of these efforts targeted community health workers, but the more health security (e.g., application of PPE material) and digital technological skills development targeted formal health professionals. The sustainability of ODA-funded HRH actions requires attention. These strategic considerations have been more the focus of HRH governance processes and monitoring reports funded via multilateral initiatives. Although the Workforce 2030 strategy and the W4H programme emphasise and advocate for long-term integrated gender-sensitive actions across the health labour market spectrum, development funded HRH actions too often follow a short programme cycle and remain isolated from the required multisectoral health sector and economic reforms. Country ownership and participation in programme design and implementation by national and local stakeholders varies. While such a programmatic commitment differs between countries and with several positive examples existing, such as in Ethiopia, Rwanda and Cambodia [5], it was for many bilateral funded HRH projects unclear to assess how their activities actually contributed to national HRH strategic development and health system reforms. A more sustainable and integrated workforce partnership and leadership is required [70]. This would need a strengthened ‘health workforce literacy’ amongst all stakeholders involved. With such a locally owned approach in place, Ministries of Finance and international donors could be approached to provide financial and technical assistance as needed [71].

### Governance, accountability and shared responsibility

This leads to a second observation. Governance and accountability between development actors and across the ten UNHEEG policy recommendations could be considerably improved. There has been only limited action for the financial enablers required to transform the workforce. The decent employment and women’s labour rights aspects are often talked about, but too little followed by concrete development programmes and funded initiatives. The logic here is that this is a domestic state responsibility that development actors can support, but cannot take long-term responsibility for. We see inconsistencies here. In a financialised and globalised international economy, many LMICs have, de facto not de jure, only limited autonomy to increase their fiscal space investing in the health employment [72]. 143 countries expect to cut public budget spending in 2023, due to economic volatility following the pandemic and other crises [47]. Health employment financing in LMICs should not be a sole domestic responsibility, but requires shared responsibility for cooperative action—as pointed out by the Joint Learning Initiative on HRH some twenty years ago [73]. It would be fair to develop a coherent global framework for health workforce finance and investment based on shared responsibility principles, including debt relief and cancelation for LICs [74]. Doing so, accountability and coordination between actors is not only needed at the national level, but also at the international level. The GHWA was originally tasked with playing a role in defining and monitoring this global health workforce agenda. Fifteen years after the Kampala declaration and agenda for Global Action [75] we unfortunately assess that, policies toward building coherent ‘global leadership for health workforce solutions’ and ‘securing additional and more productive investment in the health workforce’ have been restricted in scope and outcome [76].

### Generating evidence on relevance and effectiveness of health workforce investments

Lastly, while a broad range of HRH actions and outputs have been identified, available data on programme outcomes and especially impacts are few in number. While there is solid evidence that investment in the health workforce is relevant for economic growth [76], this impact was difficult to be proven for most of ODA-funded HRH actions found in this review. This is not surprising, given its multiple variables, including required domestic investments and commitments for HRH development, as well as external factors like the Covid-19 pandemic, social and economic instability, etc. Nevertheless, research on the actual effectiveness of HRH development action, including for numerous cross-cutting workforce-specific initiatives in global health programmes, is only limited conducted via formal academic studies.

### ODA funding for HRH interventions

Our findings do complement, and are coherent with, the quantitative analysis on ODA funding for HRH for the period 2016–2020 [3]. Despite an evident increase in funding during the Covid-19 outbreak, HRH-funded activities have changed only marginally over time as training remains the primary type of activity being supported. In 2020, 7% of ODA (US$4 billion) has been directed to HRH activities. Moreover, most of the funded activities have remained short-term in scope with limited impact on developing sustainable health systems in the long term [3]. ODA funding for HRH hence falls far short in contributing to US$278 billion needed annually for HSS in 67 LMICs for attaining the SDGs [2].In 2021, the director of WHO’s Health Workforce Department called this a *‘global scramble for health workers’* and that we need sustainable investments in education and employment: ‘*This needs multiyear long-term investments, it is a moral obligation, a billion here and a billion there is not going to make a difference.’* [54].

### A health workforce crisis momentum?

In the wake of the Covid-19 pandemic and given the rapid growing inequities in and beyond the health care sector a leap frog ambition is needed. In the WHO European region policy action is required as the ageing of the health and care workforce is a concern and poses a threat to the sustainability of the workforce due to the challenge of replacing workers when they retire [77]. The Biden–⁠Harris Administration Global Health Worker Initiative likewise realises the need to invest in health and care workers as a crucial contribution to the Sustainable Development Agenda [78]. WHO’s global health and care worker compact, adopted by the World Health Assembly in 2022 provides guidance on how to protect health and care workers and safeguard their rights, and to promote and ensure decent work [79].

While the size of the health workforce increases globally as more jobs are, and will, continue to be created in the health economy, this masks considerable inequities, particularly in WHO African and Eastern Mediterranean regions, and alarmingly among the 55 countries on the WHO Support and Safeguards List. In these two regions and countries, characterised by a growing population size and the rising demand for health services, improvement in health workforce shortages remain stagnant by 2030 without urgent policy action [80]. Moreover, the quality of these globally growing health sector jobs, by some defined as ‘care extractivism’, deserves more research from a decent employment perspective. The actual distribution of these jobs between the private and public sector may imply increasing health system inequities [40]. Research on HRH policies in post-conflict and post-crisis settings indicates the difficulty to sustain ODA investments. Health seems to be more a pre-occupation of the international community than of governments in fragile states as these may face other policy priorities [81].

### Five policy recommendations

Based on the three observations made above, the analysis of ODA funding for HRH programmes and the persistent health workforce crisis, several international policy priorities should be considered. In a policy brief targeting the G7 countries, Soucat and colleagues provide four relevant policy recommendations for developing HRH in a globalised world, arguing the need to move from an efficiency to an equity investment approach in the social and health care sector. Their recommendations are: (1) develop a truly coherent global HRH agenda, over and beyond development aid for LICs; (2) address the health skills supply gap of the global market by providing financial and non-financial incentives to middle- and high-school graduates to enter health professions; (3) significantly increase ODA funding via investments in creating health sector jobs for youth and women; and (4) create sustainable health labour mobility partnerships between countries (82). Following the analyses from the WHO Council on the Economics of Health for All a fifth recommendation can be articulated [83]; development programmes and investments by the IFIs to support (community) health workforce training and education should be structurally complemented by accommodating macro-economic policy to increase public spending for decent employment, including flexibility in fiscal deficits, expansionary monetary policy, extensive debt cancellation and the issuance of more IMF Special Drawing Rights, targeting tax avoidance and evasion by transnational cooperation [52].

## Limitations

The aim of this review has not been to evaluate the implementation of individual programmes. Given the variation of sources and heterogeneity of the data it has been impossible to compare the qualitative level of evidence between the data sources. Triangulation of different data-sources allowed for a general overview of actors involved and their main field of action. The researchers have conducted a rapid review of the secondary data available while acknowledging several inadequacies: many HRH development actions are not covered in academic literature; HRH development actions may be part of broader HSS, UHC, PHC or global health security actions; they may be part of a multiplicity of smaller initiatives across the globe often by volunteers and health professionals that receive limited renumeration for these collaborations. The macro-perspective provided in this analysis needs ideally to be complemented by primary data collection at the (sub)-national level to assess the actual sustainability of collaborative HRH actions. We likely only see the tip of the iceberg of ongoing cooperation. The researchers have conducted reviews and analyses on international HRH policies over more than a decade and this has structured, and may have impacted, their overall assessment.

## Conclusion

This exploratory analysis had provided a generic and comparative qualitative overview of ODA relevant actions in the HRH domain to attain the SDGs. More specifically, it has assessed the contribution of development actors to the implementation of WHO’s Workforce 2030 strategy and the recommendations of the UNHEEG commission. The global health and care workforce needs are much recognised, especially given the impact on the Covid-19 pandemic. Unfortunately, this has only partially led to actual HRH commitments and funded programmes by development actors. While a range of short-term training and education programmes exists, there is too little actual cooperation for sustainable and strategic health workforce development, including the creation of decent employment in the health sector. This can partly be attributed to a macro-economic environment of fiscal limitations and austerity in times of multiple crises, notably in a number of LMICs, and partly because foreign policy and (global health) development cooperation has focused on other collaborations and crisis management. Twenty years after the Joint Learning Initiative on HRH there is still an urgent need to take shared responsibility for cooperative action for overcoming the health workforce crisis.

## Data Availability

FAIR research principles are applied and data extraction sheet is available on request via the corresponding author.

## Abbreviations

Covid-19: Coronavirus disease 2019
EIB: European Investment Bank
Gavi: Gavi the Vaccine Alliance
GF: Global Fund to Fight AIDS, Tuberculosis and Malaria
GFF: Global Financing Facility
GHWA: Global Health Workforce Alliance
GHWN: Global Health Workforce Network
GIZ: German Development Cooperation agency
HCWF: Health and Care Workforce
HRH: Human Resources for Health
HSS: Health Systems Strengthening
ICRC: International Committee of the Red Cross
IFIs: International Financial Institutions
IMF: International Monetary Fund
IPHWM: International Platform on Health Workforce Mobility
JICA: Japan International Cooperation Agency
LIC: Low-income countries
LMICs: Low- and middle-income countries
MSF: Médecins Sans Frontières
NHS: National Health Service
NHWA: National Health Workforce Accounts
NGO: Non-Governmental Organisation
Norad: Norwegian Agency for Development Cooperation
ODA: Official Development Assistance
OECD: Organisation for Economic Co-operation and Development
PHC: Primary Health Care
PPE: Personal Protection Equipment
SDGs: Sustainable Development Goals
SIDA: Swedish International Development Cooperation Agency
SSA: Sub-Saharan Africa
UHC: Universal Health Coverage
UN: United Nations
UNDP: United Nations Development Programme
UNHEEG: United Nations High-Level Commission on Health Employment and Economic Growth
UK: United Kingdom
USA: United States of America
USAID: United States Agency for International Development
US$: United States dollar
W4H: Working for Health
WB: World Bank
WHO: World Health Organization
